# Relationship among *Strongyloides stercoralis* Infection, Human T-Cell Lymphotropic Virus Type 1 Infection, and Cancer: A 24-Year Cohort Inpatient Study in Okinawa, Japan

**DOI:** 10.4269/ajtmh.15-0556

**Published:** 2016-02-03

**Authors:** Teruhisa Tanaka, Tetsuo Hirata, Gretchen Parrott, Miwa Higashiarakawa, Takeshi Kinjo, Tetsu Kinjo, Akira Hokama, Jiro Fujita

**Affiliations:** Department of Infectious, Respiratory, and Digestive Medicine, Faculty of Medicine, University of the Ryukyus, Okinawa, Japan; Department of Endoscopy, University of Ryukyus Hospital, Okinawa, Japan

## Abstract

This study evaluated the prevalence of *Strongyloides stercoralis* infection and human T-cell lymphotropic virus type 1 (HTLV-1) infection in the population. In addition, this study investigated the relationship between *S. stercoralis* infection or HTLV-1 infection and a patient's risk of developing related cancers. This is a retrospective cohort study of 5,209 patients. The prevalence of *S. stercoralis* infection was 5.2% among all patients. The prevalence among men (6.3%) was significantly higher than among women (3.6%, *P* < 0.001). The prevalence of HTLV-1 infection among this population was 13.6% and the prevalence among women (15.5%) was significantly higher than that of men (12.3%, *P* < 0.001). HTLV-1 seroprevalence was higher in patients with liver cancer (*P* = 0.003, odds ratio [OR]: 1.91, 95% confidence interval [CI]: 1.24, 2.95) and in those with lymphoma other than adult T-cell leukemia/lymphoma (ATLL) (*P* = 0.005, adjusted OR: 2.76, 95% CI: 1.36, 5.62) if compared with patients without any neoplasm. The prevalence of both *S. stercoralis* and HTLV-1 in the Okinawan population has been steadily decreasing over the past 24 years. HTLV-1 infection significantly increases the odds of developing liver cancer and lymphomas other than ATLL.

## Introduction

*Strongyloides stercoralis* is one of the most common human gastrointestinal parasites in the world. The Okinawa Prefecture of Japan is located in a subtropical region, which is endemic for *S. stercoralis*.^1^,^2^ With humid and warm soil, subtropical regions provide the preferred external environment for *S. stercoralis*. The filariform larvae, which inhabit the soil, usually infect humans via skin penetration. After infection, the larvae travel to the duodenum to become adult females. Rhabditiform larvae, hatched from eggs produced by the females, are excreted from the human host. However, some larvae reinfect the host through the intestinal mucosa or perianal skin, using a process called autoinfection, which is unique to only a few parasites, allowing *S. stercoralis* to complete its life cycle and proliferate successfully within a single host.^3^

Okinawa Prefecture is also endemic for human T-cell lymphotropic virus type 1 (HTLV-1), a virus associated with adult T-cell leukemia/lymphoma (ATLL).^4^–^6^ There are three possible transmission routes for HTLV-1: sexual transmission, mother to child transmission via breast milk, and exposure to contaminated blood. In Japan, the virus is most commonly transmitted from mother to child.^7^ It is well known that infection of HTLV-1 early in life may increase the risk for subsequent diseases, particularly ATLL.^8^

Infectious agents, including parasites, often have oncogenic potential. Infection can initiate or promote carcinogenesis by any of three main mechanisms: 1) chronic inflammation due to a prolonged persistence of infectious agents within the host tissue, 2) insertion of active oncogenes into the host genome, and 3) reduced immunosurveillance as a result of immunosuppression.^9^ Similarly, the autoinfection route of *S. stercoralis* in host gastrointestinal and lung tissue also has the potential to cause chronic inflammation and promote subsequent carcinogenesis. Some studies have reported an association between HTLV-1 infection and carcinomas other than ATLL; however, this link is still controversial.^10^–^12^

With this foundational evidence, we conducted an inpatient study to investigate the prevalence of *S. stercoralis* and HTLV-1 infections, as well as the relationship between these two infections. Within the same cohort, we also conducted a retrospective cohort study to investigate the relationship between a history of *S. stercoralis* or HTLV-1 infection and a potentially increased risk of developing various cancers.

## Material and Methods

### Study population.

This retrospective cohort study included 5,209 patients (3,154 men and 2,055 women) who were admitted to the First Department of Internal Medicine for Infectious, Respiratory, and Digestive Medicine at University of Ryukyus Hospital in Okinawa between 1991 and 2014 (Table 1).

Controls, included for the investigation of *S. stercoralis* infection and its association with the development of cancer, were composed of all patients born before 1960 without cancer or a history of cancer. The controls used in the HTLV-1 infection analysis included all patients born before 1990 without cancer or a history of cancer. All patients were admitted as inpatients to the First Department of Internal Medicine at the University of Ryukyus Hospital during the same period.

### Evaluation of *S. stercoralis* and HTLV-1 infections.

Infection of *S. stercoralis* was diagnosed in all patients using the stool agar plate culture method.^13^ Serum antibody to HTLV-1 was measured in all patients using the gelatin particle agglutination method.^14^

### Cancer diagnosis.

The diagnosis of cancer was based on histology, cytology, and radiological findings. Patients diagnosed with metastatic cancer were excluded because the source of primary cancer could not be determined within reasonable time constraints.

### Statistical analyses.

The χ^2^ test was used to compare the prevalence of *S. stercoralis* or HTLV-1 infection between sexes. The χ^2^ test was also used to compare the prevalence of each cancer in a crude analysis with a history of *S. stercoralis* or HTLV-1 infection. Logistic regression analyses adjusted for age and sex were used to examine the odds of developing each cancer considering the incidence of *S. stercoralis* or HTLV-1 infection. All statistical analyses and graphical representations were performed using SPSS (version 21.0; IBM Corp., Armonk, NY) software packages. The *P* values reported here are two sided.

## Results

### Prevalence of *S. stercoralis* and HTLV-1 infection.

**Figure 1.**
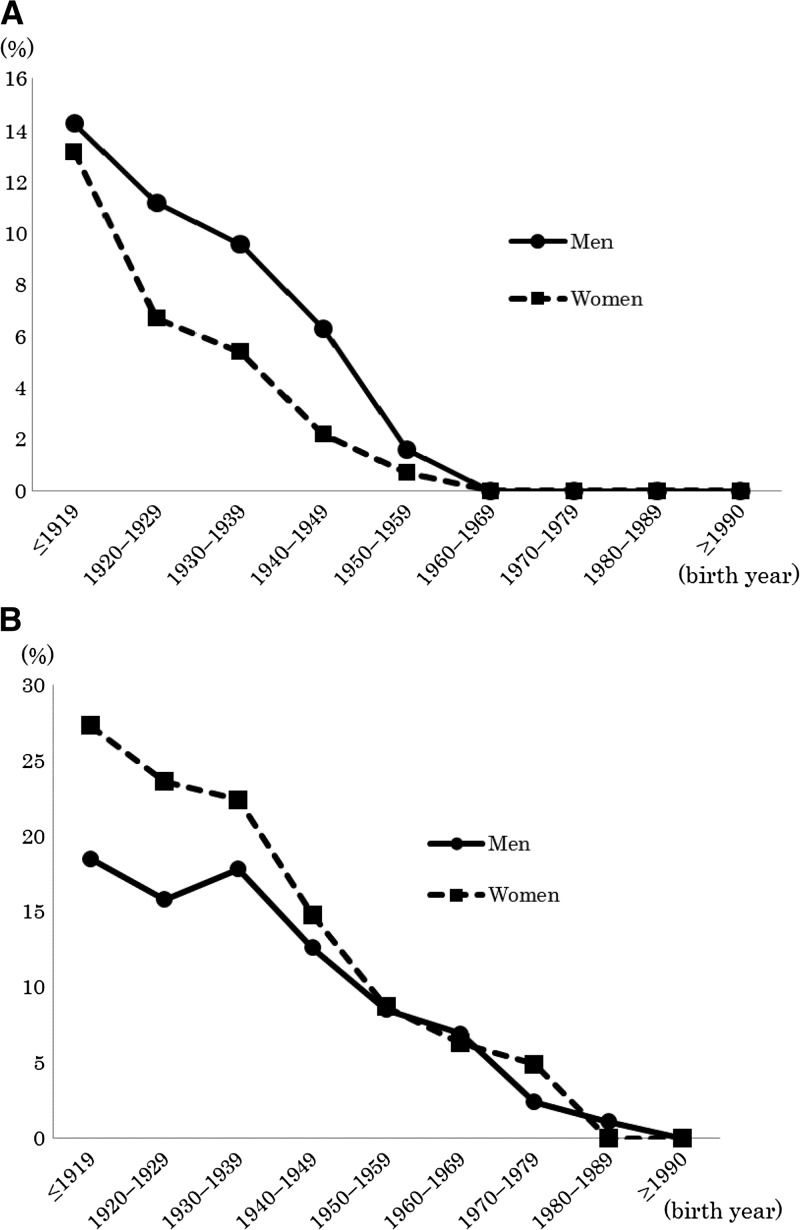
The study included 5,209 patients who were admitted to the First Department of Internal Medicine for Infectious, Respiratory, and Digestive Medicine at the University of Ryukyus Hospital in Okinawa, Japan, between 1991 and 2014. (**A**) The prevalence of *Strongyloides stercoralis* infection in men (circles) and women (squares) by age. (**B**) The prevalence of human T-cell lymphotropic virus type 1 infection in the men (circles) and women (squares) by age.

The study population was composed of 3,154 men and 2,055 women, with a mean age of 56.4 ± 17.9 (standard deviation [SD]) years (range: 11–101 years). The total prevalence of *S. stercoralis* infection in our study population was 5.2% (Table 2, Figure 1A

). The prevalence of *S. stercoralis* in the male population (6.3%) was significantly higher than that in the female population (3.6%, *P* < 0.001). There were no patients with a *S. stercoralis* infection that were born after 1960. The total prevalence of HTLV-1 infection was 13.6% (Table 2, Figure 1B). The prevalence of HTLV-1 infection in men and women was 12.3% and 15.5%, respectively. HTLV-1 infection was significantly more prevalent in women than in men (*P* < 0.001). The number of *S. stercoralis* and HTLV-1 infections steadily decreased for both sexes in each successive generation.

To evaluate the relationship between *S. stercoralis* infection and HTLV-1 infection, we compared only patients born before 1960. The total number of patients born before 1960 was 4,056 (2,459 men and 1,597 women). Within this population, the prevalence of *S. stercoralis* infection was significantly higher in patients with HTLV-1 infection compared with that in patients without HTLV-1 infection (Tables 3 and 4). The odds ratio (OR) of this comparison was higher in female patients than in male patients.

### Association between *S. stercoralis* infection and each type of cancer.

Within the 4,056 patients born before 1960, we identified 1,352 patients with diagnostically confirmed cancer. The cancer patients consisted of 953 men and 399 women, with a mean age of 67.0 ± 10.2 (SD) years. The cancer-free control group consisted of 1,446 men and 1,150 women with a mean age of 61.8 ± 12.9 (SD) years.

Table 5 presents the prevalence and association of *S. stercoralis* infection among cancer patients and controls. The prevalence of *S. stercoralis* infection in controls and in cancer patients were significantly different from one another at 5.7% and 8.7% (*P* < 0.001), respectively. Using a logistic regression model adjusted for age and sex, we calculated the OR stratified for each cancer. Although our data suggest that *S. stercoralis* patients are more likely to develop cancer (*P* < 0.001), *S. stercoralis* infection was not found to be significantly associated with any specific type of cancer.

### Association between HTLV-1 infection and cancer.

Within the 5,168 patients born before 1990, we identified 1,437 patients with diagnostically confirmed cancer. In this population, the cancer patients consisted of 1,005 men and 432 women with a mean age of 65.4 ± 11.9 (SD) years. The control group consisted of 2,056 men and 1,556 women with a mean age of 51.4 ± 17.9 (SD) years.

Table 6 presents the prevalence and association between HTLV-1 infection among controls and cancer patients. The prevalence of HTLV-1 infection in controls and in cancer patients were significantly different from one another at 12.9% and 15.2% (*P* = 0.03), respectively. In addition, the prevalence of HTLV-1 infection in patients with liver cancer (*P* = 0.01) or with lymphomas other than ATLL were significantly higher than that in patients with other types of cancer (*P* = 0.03). Using a logistic regression model adjust for age and sex, we calculated the OR stratified for each cancer. HTLV-1 infection was not shown to significantly increase the odds of developing most types of cancer, except for liver cancer and lymphomas other than ATLL. Patients with an HTLV-1 infection in our cohort were approximately twice as likely to develop liver cancer (OR: 1.91, 95% confidence interval [CI]: 1.24, 2.95) and approximately three times more likely to develop lymphoma other than ATLL (OR: 2.76, 95% CI: 1.36, 5.62) compared with patients without HTLV-1.

## Discussion

Our results show that there were no patients born after 1960 with *S. stercoralis* infection in our cohort. Although some publications report younger patients with *S. stercoralis* infection who have never traveled outside of Japan,^15^,^16^ the overall prevalence of *S. stercoralis* infection has markedly decreased since 1960. This change is most attributed to improvements in public health and sanitation. After World War II, intestinal parasitic infections were common in Okinawa because of poverty, poor sanitation, the use of human waste as fertilizer, and the common practice of barefoot agricultural work.^15^ At that time, public health centers also lacked the ability to detect, treat, or provide prevention for parasites.^17^ In Ozato village, Okinawa, in 1957 the recorded prevalences of hookworm and *S. stercoralis* infections were 78.9% and 10.3%, respectively.^17^ After implementation of the “Zero Parasite Campaign” from 1965 to 1969, the infection rate of parasites was drastically reduced and soil sanitation was improved.

Our study also shows that the prevalence of HTLV-1 infection is decreasing steadily, which supports existing literature from Japan and Okinawa.^18^,^19^ Satake and others suggested this reduction might be called the “birth cohort effect” whereby the high-prevalence cohort (those born 1930–1960) ages while younger cohorts (those born after 1960) have lower prevalence rates.^20^ These findings may be the result of increased knowledge regarding HTLV-1 and its transmission routes.^18^ In Japan, the transmission of virus via transfusion has been eliminated since the implementation of HTLV-1 screening of donated blood in 1986. Japanese mothers have increased the number of bottle-fed babies,^21^–^23^ thereby decreasing the vertical infection of HTLV-1. In 2011, the Japanese Ministry of Health, Labour and Welfare initiated a nationwide program to prevent mother-to-child infection by screening all pregnant women for HTLV-1 infection and recommending bottle feeding for women with positive results.^24^

The data suggest a strong correlation between *S. stercoralis* and HTLV-1 infections. The prevalence of *S. stercoralis* infection was significantly higher (*P* < 0.001) in patients with HTLV-1 infection compared with that in patients without HTLV-1 infection. Patients infected with HTVL-1 developed *S. stercoralis* infection 2.4 times more often than noninfected patients. Multiple studies in Okinawa have showed an increased risk for *S. stercoralis* infection when the host is immunocompromised,^19^,^25^,^26^ and similar findings were reported in studies conducted in other regions, such as South America.^27^–^29^ Furthermore, when the data were stratified for sex, we also found that females were four times more likely to have concurrent infections of *S. stercoralis* and HTLV-1. This altered susceptibility is most likely due to the difference in effectiveness of HTLV-1 transmission. It has been documented that male-to-female sexual transmission is more efficient than female-to-male sexual transmission.^30^ Sexual transmission requires intimate and prolonged contact between partners.^31^ Several studies have also suggested a correlation between older age and risk of infection, particularly for women, whose increased susceptibility may be due to the thinning of vaginal epithelia tissue after menopause.^30^,^32^,^33^ However, some studies have shown that there are no correlations between the prevalence of *S. stercoralis* and HTLV-1 infections.^27^,^34^ Carvalho and others suggested that the controversial results were due to the type of technique used to determine *S. stercoralis* infection: stool examination or serological test.^27^ In our study, results show a strong correlation between *S. stercoralis* and HTLV-1 infections because only stool examinations were used for determining *S. stercoralis* infection.

No statistically significant associations between *S. stercoralis* infection and the development of any specific types of cancer were found in our data. One study from Okinawa shows a significantly high prevalence of *S. stercoralis* infection in patients with biliary tract cancer.^35^ Adult *S. stercoralis* persist in human duodenum and upper jejunum, and the nematodes often migrate via the biliary tract. The resulting damage could cause cholangitis or pancreatitis or it could initiate and promote carcinogenesis.^36^–^39^ Although our study shows that patients with biliary tract cancer may be almost twice as likely to have evidence of *S. stercoralis* infection as control patients (OR: 1.90, 95% CI: 0.93, 3.87), the evidence for this association is not statistically significant (*P* = 0.08). This result may be due to low statistical power, as only 69 cases of biliary tract cancers were included in our cohort.

Some studies suggest that HTLV-1 infection is associated with many types of cancer, mainly liver and other blood cancers.^12^,^40^–^42^ Other reports showed that HTLV-1 infection may have a protective effect against gastric cancers.^10^,^11^,^43^ Our data show that HTLV-1 infection is not associated with cancer development apart from liver cancer and lymphomas other than ATLL. In addition, although our study found that patients with gastric cancer might be less likely to have evidence of HTLV-1 infection than patients with other types of cancer (OR: 0.75, 95% CI: 0.50, 1.12), the data are not statistically significant (*P* = 0.16). Similarly, we saw a trend that patients with esophageal cancer might be less likely to have evidence of HTLV-1 infection than patients with other types of cancer (OR: 0.56, 95% CI: 0.29, 1.11), but this difference also failed to reach statistical significance (*P* = 0.10). A report from Iran also described a trend toward an association of HTLV-1 infection and esophageal squamous cell carcinoma, but their data similarly failed to reach statistical significance.^44^

This study found that HTLV-1 infection is associated with the development of liver cancer (OR: 1.91, 95% CI: 1.24, 2.95, *P* = 0.003). Similarly, a previous report showed a high association of HTLV-1 infection with the incidence of liver cancer.^12^ Here, we also showed that HTLV-1 infection in patients with non-ATLL lymphoma was significantly higher than that in patients with other types of cancer (OR: 2.76, 95% CI: 1.36, 5.62, *P* = 0.005). Although HTLV-1 has not been previously associated with the occurrence of lymphoma other than ATLL, some reports have suggested that HTLV-1 carriers with B-cell lymphoma tend to have worse prognosis or that the frequency of primary malignant neoplasms in HTLV-1 carriers is higher than that in seronegative cases.^40^,^41^ Another report also suggested that the interaction between Epstein–Barr virus and HTLV-1 could promote T- and B-cell dysfunctions and cell proliferation and inhibit apoptosis, favoring lymphomagenesis.^42^

Some limitations exist in this study. First, only the patients that were admitted to the Department of Infectious, Respiratory, and Digestive Medicine University of the Ryukyus Hospital and tested for HTLV-1 or *S. stercoralis* were included. The use of this population may introduce a selection bias in our results. Second, we did not examine the effect of confounding variables in our logistic regression, including other known carcinogens, such as smoking, drinking, parasitic infections other than *S. stercoralis*, and viral infections other than HTLV-1 (hepatitis B/C virus, Epstein–Barr virus, etc.). All patients with HTLV-1 carrier status were included in this study regardless of age. The number of young patients that were included in the HTLV-1-associated cancer development sub-analysis may have skewed the results in the opposite direction. To help normalize the results, age and sex were included in the logistic regression model to eliminate those biases.

## Conclusions

Our study indicates that the prevalence of *S. stercoralis* and HTLV-1 infections have been decreasing in recent years. *Strongyloides stercoralis* infection was 2.4 times more likely in patients with HTLV-1 infection than in patients without it. Diligence toward the prevention of these diseases through decreased poverty and increased sanitation has proven effective. Continued improvements in education, testing, and treatment could easily eliminate *S. stercoralis* infections and drastically reduce the prevalence of HTLV-1 infections.

In addition, HTLV-1 infection in patients with hepatic cancer or lymphomas other than ATLL appears to be significantly higher than that in patients with other types of cancer. Further investigation regarding the possible mechanisms behind these associations is needed.

## Figures and Tables

**Table 1:** Patient characteristics (*N* = 5,209)

Men	3,154 (60.5%)
Age	56.4 (SD: 17.9) range: 11–101 years
Cancers	
Esophagus	114 (2.2%)
Stomach	262 (5.0%)
Biliary tract	71 (1.4%)
Liver	143 (2.7%)
Colon and rectum	200 (3.8%)
Lung	444 (8.5%)
Pancreas	38 (0.7%)
Lymphoma without ATLL	42 (0.8%)
Others*	171 (3.3%)

ATLL = adult T-cell leukemia/lymphoma; SD = standard deviation.

* Other cancers include breast cancer, uterine cancer, kidney cancer, pharyngeal and laryngeal cancer, and ATLL, among others.

**Table 2:** Prevalences of *Strongyloides stercoralis* infection and HTLV-1 infection

Birth year	Number of *S. stercoralis*-positive patients/number of tested patients (%)			Number of HTLV-1 positive patients/number of tested patients (%)		
	Men	Women	Total	Men	Women	Total
≤ 1919	24/168 (14.8)	14/106 (13.2)	38/274 (13.9)	31/168 (18.5)	29/106 (27.3)	60/274 (21.9)
1920–1929	59/526 (11.2)	22/326 (6.7)	81/852 (9.5)	83/526 (15.8)	77/326 (23.6)	160/852 (18.8)
1930–1939	76/794 (9.6)	27/500 (5.4)	103/1,294 (8.0)	141/794 (17.8)	112/500 (22.4)	253/1,294 (19.6)
1940–1949	33/522 (6.3)	8/365 (2.2)	41/887 (4.6)	66/522 (12.6)	54/365 (14.8)	120/887 (13.5)
1950–1959	7/449 (1.6)	2/300 (0.7)	9/749 (1.2)	38/449 (8.5)	26/300 (8.7)	64/749 (8.5)
1960–1969	0/331 (0.0)	0/207 (0.0)	0/538 (0.0)	23/331 (6.9)	13/207 (6.3)	36/538 (6.7)
1970–1979	0/251 (0.0)	0/163 (0.0)	0/414 (0.0)	6/251 (2.4)	8/163 (4.9)	14/414 (3.4)
1980–1989	0/88 (0.0)	0/72 (0.0)	0/160 (0.0)	1/88 (1.1)	0/72 (0.0)	1/160 (0.6)
≥ 1990	0/25 (0.0)	0/16 (0.0)	0/41 (0.0)	0/25 (0.0)	0/16 (0.0)	0/41 (0.0)
Total	199/3,154 (6.3)*	73/2,055 (3.6)	272/5,209 (5.2)	389/3,154 (12.3)	319/2,055 (15.5)*	708/5,209 (13.6)

HTLV-1 = human T-cell lymphotropic virus type 1.

* *P* < 0.001 for male vs. female by χ^2^ analysis.

**Table 3:** Crude analysis (all cases and controls born before 1960)

		HTLV-1		
		Positive	Negative	Total
*Strongyloides stercoralis*	Positive	82 (2.0%)	190 (4.7%)	272
	Negative	575 (14.2%)	3,209 (79.1%)	3,784
	Total	657	3,399	4,056

HTLV-1 = human T-cell lymphotropic virus type 1.

Odds ratio = 2.41 (95% confidence interval = 1.83, 3.17; *P* < 0.001) by χ^2^ analysis.

**Table 4:** Gender-stratified analyses

		HTLV-1		
		Positive	Negative	Total
Men*				
*Strongyloides stercoralis*	Positive	48 (2.0%)	151 (6.1%)	199
	Negative	311 (12.6%)	1,949 (79.3%)	2,260
	Total	359	2,100	2,459
Women†				
		
*S. stercoralis*	Positive	34 (2.1%)	39 (2.4%)	73
	Negative	264 (16.5%)	1,260 (78.9%)	1,524
	Total	298	1,299	1,597

HTLV-1 = human T-cell lymphotropic virus type 1.

* Odds ratio (OR) = 1.99 (95% confidence interval (CI) = 1.41, 2.82; *P* < 0.001) by χ^2^ analysis.

† OR = 4.16 (95% CI = 2.58, 6.72; *P* < 0.001) by χ^2^ analysis.

**Table 5:** Association between *Strongyloides stercoralis* infection and each cancer type (patients born before 1960, *N* = 4,056)

	Stratified analysis		Multivariate analysis		
	*S. stercoralis* infection rate	*P* value	OR	95% CI	*P* value
Control	5.7% (147/2,596)				
Total cancer	8.7% (117/1,352)	< 0.001*	1.28	0.98–1.66	0.06‡
Esophagus	6.4% (7/109)	0.48†	0.65	0.29–1.45	0.29§
Stomach	9.9% (24/242)	0.45†	1.22	0.76–1.97	0.42§
Biliary tract	14.5% (10/69)	0.05†	1.90	0.93–3.87	0.08§
Liver	6.4% (9/140)	0.43†	0.72	0.35–1.47	0.37§
Colon and rectum	7.7% (15/194)	0.68†	0.94	0.53–1.66	0.82§
Lung	9.6% (40/418)	0.46†	1.09	0.73–1.64	0.68§
Pancreas	5.4% (2/37)	0.77†	0.83	0.19–3.55	0.80§
Lymphoma without ATLL	2.7% (1/37)	0.37†	0.28	0.28–2.08	0.21§

ATLL = adult T-cell leukemia/lymphoma; CI = confidence interval; OR = odds ratio.

* A χ^2^ analysis was used to compare *S. stercoralis* infection between patients with cancer (total cancer) and control patients (control).

† A χ^2^ analysis was used to compare *S. stercoralis* infection between patients with each type of cancer and patients with other types of cancer.

‡ A logistic regression analysis, adjusted for age and sex, was used to compare *S. stercoralis* infection between patients with cancer (total cancer) and control patients (control).

§ A logistic regression analysis, adjusted for age and sex, was used to compare *S. stercoralis* infection between patients with each type of cancer and patients with other types of cancer.

**Table 6:** Association between HTLV-1 infection and each cancer type (patients born before 1990, *N* = 5,168)

	Stratified analysis		Multivariate analysis		
	HTLV-1 infection rate	*P* value	OR	95% CI	*P* value
Control	12.9% (467/3,612)	–	–	–	–
Total cancer	15.2% (219/1,437)	0.03*	0.90	0.75–1.09	0.28‡
Esophagus	8.8% (10/114)	0.06†	0.56	0.29–1.11	0.10§
Stomach	12.2% (32/262)	0.15†	0.75	0.50–1.12	0.16§
Biliary tract	16.9% (12/71)	0.73†	0.96	0.53–1.84	0.90§
Liver	22.3% (32/143)	0.01†	1.91	1.24–2.95	0.003§
Colon and rectum	15.0% (30/200)	1.00†	0.91	0.60–1.40	0.68§
Lung	13.5% (60/444)	0.23†	0.81	0.58–1.12	0.19§
Pancreas	7.8% (3/38)	0.26†	0.45	0.14–1.49	0.19§
Lymphoma without ATLL	28.5% (12/42)	0.03†	2.76	1.36–5.62	0.005§

ATLL = adult T-cell leukemia/lymphoma; HTLV-1 = human T-cell lymphotropic virus type 1; CI = confidence interval; OR = odds ratio.

* A χ^2^ analysis was used to compare HTLV-1 infection between patients with cancer (total cancer) and control patients (control).

† A χ^2^ analysis was used to compare HTLV-1 infection between patients with each type of cancer and patients with other types of cancer.

‡ A logistic regression analysis, adjusted for age and sex, was used to compare HTLV-1 infection between patients with cancer (total cancer) and control patients (control).

§ A logistic regression analysis, adjusted for age and sex, was used to compare HTLV-1 infection between patients with each type of cancer and patients with other types of cancer.
